# An Engineered Approach to Stem Cell Culture: Automating the Decision Process for Real-Time Adaptive Subculture of Stem Cells

**DOI:** 10.1371/journal.pone.0027672

**Published:** 2011-11-16

**Authors:** Dai Fei Elmer Ker, Lee E. Weiss, Silvina N. Junkers, Mei Chen, Zhaozheng Yin, Michael F. Sandbothe, Seung-il Huh, Sungeun Eom, Ryoma Bise, Elvira Osuna-Highley, Takeo Kanade, Phil G. Campbell

**Affiliations:** 1 Department of Biological Sciences, Carnegie Mellon University, Pittsburgh, Pennsylvania, United States of America; 2 Department of Biomedical Engineering, Carnegie Mellon University, Pittsburgh, Pennsylvania, United States of America; 3 Department of Computer Science, Carnegie Mellon University, Pittsburgh, Pennsylvania, United States of America; 4 Institute for Complex Engineered Systems, Carnegie Mellon University, Pittsburgh, Pennsylvania, United States of America; 5 Robotics Institute, Carnegie Mellon University, Pittsburgh, Pennsylvania, United States of America; 6 Intel Labs, Pittsburgh, Pennsylvania, United States of America; 7 Dai Nippon Printing, Tokyo, Japan; Stem Cell Research Institute, Belgium

## Abstract

Current cell culture practices are dependent upon human operators and remain laborious and highly subjective, resulting in large variations and inconsistent outcomes, especially when using visual assessments of cell confluency to determine the appropriate time to subculture cells. Although efforts to automate cell culture with robotic systems are underway, the majority of such systems still require human intervention to determine when to subculture. Thus, it is necessary to accurately and objectively determine the appropriate time for cell passaging. Optimal stem cell culturing that maintains cell pluripotency while maximizing cell yields will be especially important for efficient, cost-effective stem cell-based therapies. Toward this goal we developed a real-time computer vision-based system that monitors the degree of cell confluency with a precision of 0.791±0.031 and recall of 0.559±0.043. The system consists of an automated phase-contrast time-lapse microscope and a server. Multiple dishes are sequentially imaged and the data is uploaded to the server that performs computer vision processing, predicts when cells will exceed a pre-defined threshold for optimal cell confluency, and provides a Web-based interface for remote cell culture monitoring. Human operators are also notified via text messaging and e-mail 4 hours prior to reaching this threshold and immediately upon reaching this threshold. This system was successfully used to direct the expansion of a paradigm stem cell population, C2C12 cells. Computer-directed and human-directed control subcultures required 3 serial cultures to achieve the theoretical target cell yield of 50 million C2C12 cells and showed no difference for myogenic and osteogenic differentiation. This automated vision-based system has potential as a tool toward adaptive real-time control of subculturing, cell culture optimization and quality assurance/quality control, and it could be integrated with current and developing robotic cell cultures systems to achieve complete automation.

## Introduction

The use of stem cells for *in vitro* models of biological processes or for *in vivo* cell-based therapies typically requires total initial cell numbers that exceed those normally available from a single isolate of primary cells [1], [2], [3], [4], [5], [6]. To produce sufficient numbers of cells requires first inducing proliferation *in vitro* utilizing standard subculturing processes whereby cells undergoing proliferation in each culture vessel are periodically subdivided and re-plated into multiple vessels through several passages [1]. The decision on when to passage cells is currently based on a human operator's visual assessment of cell confluency, which refers to the amount of space in a tissue culture vessel that is occupied by cells and reflects cell population density. Predetermined schedules of time-points for subculturing might be sufficient for growing well characterized, established cell lines [7], [8], [9], [10]. However, in general, unpredictable changes or disturbances in culture conditions [11] or large variations in isolate-to-isolate applications of primary cells [12], [13] dictate that subculture be adaptively determined on-the-fly by direct observation of confluence over time [14]. Traditionally, human operators manually estimate confluence by microscopic observations and subsequently decide on the appropriate time for performing subculture. Presently, the majority of automated or semi-automated cell culture systems that are commercially available or in development still rely on either human oversight or a pre-determined schedule to monitor cell cultures [7], [8], [9], [10], [14]. While there are systems that use electrical impedance measurements of the cell-substrate as an indirect but automatic measure of confluence [15], some human oversight will still likely be required to monitor the process, including observing cell density and morphology to ensure optimal culture quality. The use of human operators to make decisions on subculturing is highly subjective and prone to intra- and inter-operator variability [7]. And, in the production of clinical-grade cells, the high cost of skilled labor substantially increases the costs of quality control (QC) and quality assurance (QA) operations [16]. Furthermore, it is not practical or cost-effective for human operators to manually observe and monitor cell cultures continously, and therefore key events such as the optimal times to perform subculture or identify problems might be missed. Delayed subculturing can result in cell overgrowth, which leads to loss of stem cell differentiative potential or stemness [11], [17], whereas premature subculturing can lead to longer production times to achieve targeted cell yields, with associated added costs. The overall lack of reproducibility and control of clinical-grade cell expansion processes is a major concern of government regulatory bodies since this has a direct impact on product performance and product reproducibility [18], [19], [20]. In addition, the lack of subculture standardization and reproducibility hampers scaled, robust and cost-effective manufacture of cells and has been cited as a major hurdle in the development of stem cell engineered products [7], [16].

Therefore, whether using a manual or robotic cell culture system, there is a need to automate monitoring of and decision-making for the subculturing process [16]. To begin to address this need, machine vision technology has been applied to detect cells and measure confluence to determine the appropriate time to culture cells [17], [21]; however, the images derived from this system are similar to that of a brightfield microscope and as such, of low-contrast [21], making it difficult to verify cell detection performance. Additionally, this system did not incorporate real-time predictive modeling of cell growth, and lacks the capability to function as part of a QA/QC system by raising warning alarms if growth was not progressing as expected and, in manually operated systems, as a tool to alert human operators in a timely manner to make preparations for subculture.

Herein we report on a new technology platform for continuous, fully automated monitoring, analysis, and predictive growth modeling of phase-contrast time-lapse microscopy imaging of the subculturing process. This platform is based on our previously developed real-time, computer vision-based cell tracking system [22], which is capable of tracking all cells and their lineages in an image sequence at high levels of confluence with high levels of accuracy and is amenable to analysis with population growth modeling tools [23]. These components are combined within the framework of a Web-based human-computer interface whereby images are acquired at 5 minute-intervals and uploaded to a server for image processing and analysis to predict future confluency. These results can be viewed over the Web, allowing human operators to conveniently monitor the process remotely. Furthermore, the system alerts the operators by email and text messaging 4 hours prior to reaching a pre-defined confluency threshold so that preparations for cell culture can be made, and an additional reminder is sent when the predefined threshold for confluency is reached. This system was validated by directing the expansion of mouse C2C12 cells as a paradigm stem cell population with the criterion that confluency must not exceed 0.5 (50%) in order to minimize the incidence of myoblast fusion that would otherwise deplete the stem cell population. Subsequently, C2C12 cells were differentiated towards myogenic and osteogenic fates to confirm that cells retained their capacity to differentiate into multiple cell types following cell expansion.

## Materials and Methods

### Cell Culture

Mouse C2C12 cells (ATTC, Manassas, VA) were grown in Dulbecco's Modified Eagle's Media (DMEM; Invitrogen, Carlsbad, CA), 10% fetal bovine serum (Invitrogen, Carlsbad, CA) and 1% penicillin-streptomycin (PS; Invitrogen, Carlsbad, CA). For myogenic differentiation, cells were grown in low serum containing myogenic differentiation media (DMEM, 2% Heat-inactivated horse serum, 1% PS) for 4 days with media renewal every 48 hours. For osteogenic differentiation, cells were grown in complete serum containing 100 ng/mL BMP-2 (Genetics Institute, Cambridge, MA) for 4 days with media renewal every 48 hours. Cells were kept at 37°C, 5% CO_2_ in a humidified incubator.

### Phase-Contrast Time-Lapse Microscopy

Time-lapse phase-contrast microscopy was performed using a Zeiss Axiovert T135V microscope (Carl Zeiss Microimaging, Thornwood, NY) equipped with a 5X, 0.15 N.A. phase-contrast objective, a custom-stage incubator capable of housing up to four 35 mm Petri dishes, and InVitro software 3.2 (Media Cybernetics Inc., Bethesda, MD). Three fields of view representative of the cell density from each dish were selected, resulting in a total of 12 fields of view per culture experiment for both human- and computer-directed subculture experiments. Microscope images contained 1392×1040 pixels with a resolution of 1.3 µm/pixel. Images were acquired at a frequency of every 5 minutes over a course of 1.5–3 days as determined by either the human operator or computer-generated confluency measurements.

### Confluency Measurement and Evaluation

Every 5 minutes, a set of phase-contrast microscope images was acquired (12 different fields of view from 4 Petri dishes) and the images were automatically uploaded from the local microscope computer to a server (Open Cirrus^™^, a HP/Intel/Yahoo! Open Cloud Computing Research Testbed, https://opencirrus.org/) via a fast and versatile file copying tool known as rsync [24], which is available at http://www.samba.org/ftp/rsync/rsync.html. The server also contained the computer vision-based cell tracking software, which segmented each image to identify and label cell and non-cell regions [25].

Confluency for each image was calculated by dividing the number of pixels labeled as ‘cells’ over the total number of pixels in the image. The overall confluency was then calculated by averaging the confluency values from an entire image set (12 images) acquired at a single time point. To evaluate the accuracy of the algorithm, 3 phase-contrast images containing cells at different levels of confluency (low, medium and high) were printed onto a piece of paper and manually segmented using a red marker pen to trace the outline of cells. These cell tracings were then digitized using a Hewlett Packard Scanjet 5550c flatbed scanner (Hewlett Packard, Palo Alto, CA). Then, the cell tracings were selected using the ‘Color Range’ tool in Adobe Photoshop 7.0 (Adobe Systems, San Jose, CA) and manually filled in with color using the ‘Paintbrush’ tool to generate a binary image consisting of cell and non-cell regions. Where necessary, a combination of the ‘Paintbrush’ and ‘Eraser’ tools was used to touch up the images because the scanner did not accurately capture the cell tracings. Pixels that contained cells in both the manually segmented and computer segmented images were considered true positives. Pixels that contained cells in the computer segmented images but did not overlap with cell positive regions in the manually segmented images were considered false positives. Pixels that contained cells in the manually segmented images but did not overlap with cell positive regions in the computer segmented images were considered false negatives. Pixels in the manually segmented images that did not contain any cells were considered true negatives. ‘Precision’ was defined as true positives divided by the sum of true positives and false positives. ‘Recall' was defined as true positives divided by the sum of true positives and false negatives.

### Confluency Prediction and Notification

The algorithm for confluency prediction was written in Matlab® (Mathworks, Natick, MA) and ran on the server, which is part of the HP/Intel/Yahoo! 's Open Cirrus^™^ Project. For the server, a virtual machine running a dual core processor and 2 GB Ram was created. For each set of images, the average confluency for the previous 300 frames was fitted to a 2^nd^ order polynomial curve using the Matlab® polyfit() function and the estimated confluency in the next 4 hours was determined using the Matlab® polyval() function. If the value of the estimated confluency within the next 4 hours was greater than or equal to the threshold for confluency (0.5), the Matlab® sendmail() function was used in conjunction with Matlab® TxtMsgCreate to send an email and text message via a Gmail (Google Inc., Mountain View, CA) simple mail transfer protocol (SMTP) server. Similarly, a reminder email and text message was also sent when the threshold for confluency had been reached.

### Cell Calculator User Interface Tool

The web calculator interface was developed using HTML and Javascript to facilitate calculation of the theoretical cell yield. At the beginning of the experiment, the target number of cells and the initial number of dishes were entered into the calculator by the user. Subsequently, during cell passaging, the average number of cells obtained per dish was obtained using a hemocytometer and this number was entered into the web calculator. If the total number of cells harvested was more than or equal to the target number of cells, a ‘Stop experiment’ message was displayed and broadcast to signal that the experiment should be terminated. Otherwise, a ‘Continue experiment’ message was issued and the user replated the cells at low density into additional Petri dishes for further cell expansion until the target cell number was achieved. For these experiments, a subculture ratio of 1∶8 was assumed.

### Real-time Adaptive Culture

C2C12 cells were seeded at a density of 1.5×10^4^ cells/35 mm dish (∼0.156×10^4^ cells/cm^2^) and allowed to attach for 3–6 hours prior to initiating phase-contrast time-lapse microscopy. Cells were cultured until an average confluency of 0.5 was reached in 4 dishes as determined by either the human operator or computer-generated confluency measurements. Both human- and computer-directed subculture observations were limited to 12 fields of view per culture experiment. At ∼0.5 confluency, the cells were trypsinized and the total number of cells were manually determined with the aid of a hemocytometer. This number was entered into the web-based cell calculator. If the total number of cells was less than the target number of cells, the experiment was continued and a serial culture of cells was plated for a subsequent round of cell expansion with monitoring of cultures via phase-contrast time-lapse microscopy. For each serial culture, a subculture ratio of 1∶8 was assumed. The experiment was terminated when the total cell number was equal to or more than the target number of cells. Following this, the differentiation capability of this expanded population of cells were tested by growing these under osteogenic and myogenic conditions and subsequently performing staining for osteogenic (Alkaline phosphatase; ALP), myogenic (Myogenin) and pluripotency (Pax7) markers.

### Alkaline Phosphatase (ALP) Staining

Cells were seeded into 12 well plates at a density of 12×10^4^ cells/well or 3.16×10^4^ cells/cm^2^ under osteogenic and control conditions for 4 days. Cells were washed in PBS and fixed for 2 min in 3.7% formaldehyde. ALP activity was detected according to the manufacturer's instructions (Kit 86C, Sigma-Aldrich, St. Louis, MO). Where required, ALP-stained images were converted to CMYK format since this color format is representative of reflected light colors as opposed to emitted light colors (RGB). Since cyan and magenta form the color blue, these channels were added together and inverted. The ‘levels’ tool and the ‘histogram’ tool in Adobe Photoshop 7.0 were used to normalize the background and determine the average pixel intensity, respectively. Statistical analysis was performed as described below.

### Myogenin and Pax7 Immunofluorescence Staining

Cells were seeded into 35 mm glass-bottom Petri dishes at a density of 30×10^4^ cells/dish or 3.16×10^4^ cells/cm^2^ under myogenic conditions for 4 days. Cells were washed in PBS, fixed in methanol for 5 min, air-dried and blocked with 10% donkey serum (Jackson Immunoresearch, West Gove, PA) for 20 min at room temperature (RT). For mouse-on-mouse staining, an additional blocking step was performed by incubating cells with 100 µg/mL donkey anti-mouse FAB (Jackson Immunoresearch, West Gove, PA) for 1 h at RT. Cells were then rinsed with wash buffer (PBS, 0.1% BSA) and incubated with primary antibodies: rabbit anti-myogenin (2 µg/mL; Santa Cruz Biotechnology Inc, Santa Cruz, CA) and mouse anti-Pax7 (2 µg/mL; Santa Cruz Biotechnology Inc, Santa Cruz, CA) overnight at 4°C. Cells were then rinsed 3 times with wash buffer and incubated with secondary antibodies for 1 h at RT – donkey anti-mouse Dylight 488 nm and donkey anti-rabbit Dylight 649 (15 µg/mL each; Jackson Immunoresearch, West Gove, PA). Lastly, cells were rinsed 5 times with wash buffer and imaged using a Zeiss Axiovert 200 M microscope (Carl Zeiss Microimaging, Thornwood, NY) equipped with a Colibri LED light source.

### Statistical Analysis

A student's t-test was performed using Microsoft Excel software (Microsoft Corporation, Redmond, WA) to determine significance among treatment groups. A *p*-*value*≤0.05 was considered statistically significant.

## Results

### Real-time Adaptive Subculture System

**Figure 1:**
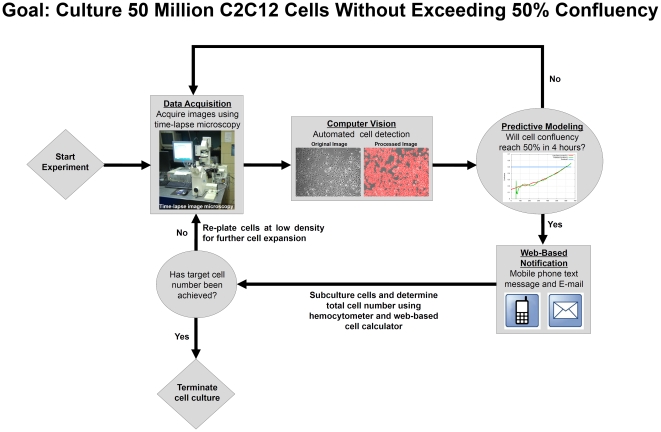
Overall Scheme of Real-Time Adaptive Cell Culture System. Data are acquired using phase-contrast time-lapse microscopy and sent to a server for image processing where the confluency is calculated and predicted. 4 hours prior to reaching a predefined threshold for confluency (0.5 confluence), an email and text message is sent to alert the user to prepare for subculture. When the cells have achieved the threshold for confluency, another email and text message is sent to remind the user. The cells are subcultured and the total cell number is counted. If the target cell number has been achieved, the experiment is terminated. Otherwise, the cells are replated at low density for further cell expansion and the process is repeated until the target cell number is achieved.

**Figure 2:**
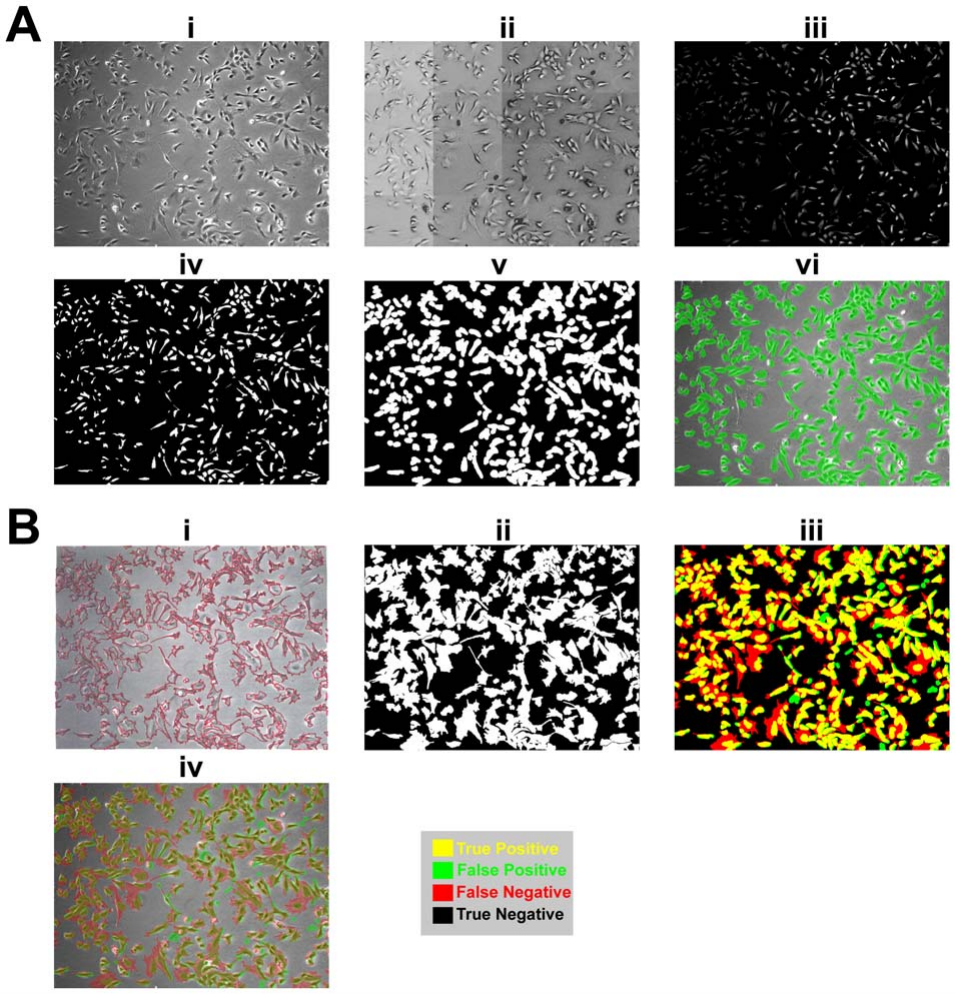
Calculation and Evaluation of Confluency. (A) Process of computer-generated confluency. (i) Original image. (ii) Image is inverted during the initial part of the restoration process to remove halo and shade-off artifacts. (iii) After restoration. (iv) Basic thresholding is applied to obtain a confluency mask. (v) The confluency mask is dilated to capture cellular processes that are hard to discern from background. (vi) The computer-generated confluency mask (green) is overlaid on top of the original image. (B) Evaluation of computer-generated confluency versus human-generated confluency. (i) Human-generated cell tracing. (ii) The cell tracing is digitized and filled in to generate a confluency mask. (iii) The computer-generated confluency mask (green) is overlaid on top of the human-generated confluency mask (red) with overlapping regions (yellow, true positive) against the background (black, true negative). Green-only regions (false positive). Red-only regions (false negative). (iv) The computer- (green) and human-generated (red) confluency masks are overlaid on top of the original image with overlapping regions highlighted (yellow).

**Figure 3:**
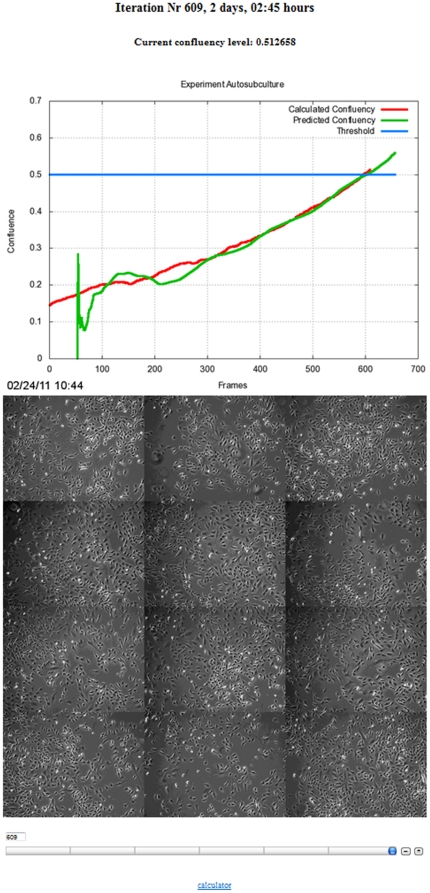
Remote Monitoring of Confluency and Predictive Modeling via the Internet. A screen capture of the graphic user interface from the real-time adaptive cell culture system illustrating the calculated confluency level and predictions (top) and individual fields of view (bottom).

**Figure 4:**
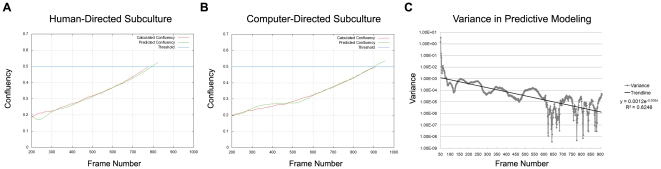
Confluency Prediction. (A) Human-directed subculture. Confluency prediction was based on the human operator's previous cell culture experience. The graphs show archived time- lapse image data from the human-directed subculture that was processed with the confluency prediction model for the purpose of comparison with the computer-directed subculture. (B) Computer-directed subculture. The predefined threshold for confluency (blue line). The calculated confluency (red line). The predicted confluency (green line). 1 frame is equivalent to 5 minutes. (C) Variance in confluency prediction and actual confluency measurement in computer-directed subculture. Variance (grey line). Trendline (black line).

**Figure 5:**
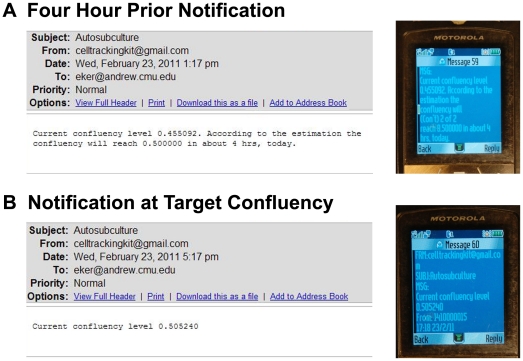
Email and Text Notification. 4 hours prior to reaching the predefined threshold for confluency, an email and text message is sent to alert the human user to prepare for subculture. Once the threshold for confluency is reached, a reminder email is sent.

**Figure 6:**
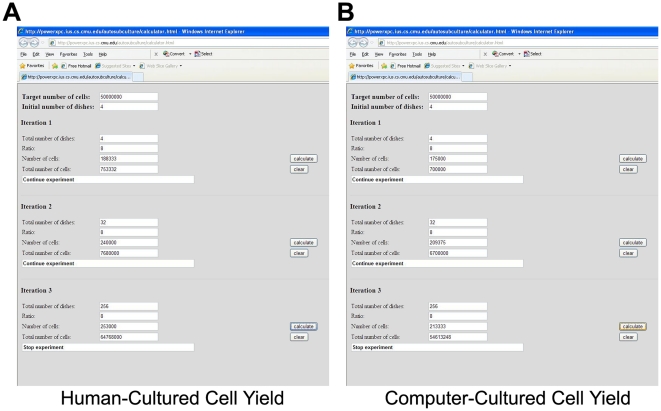
Total Theoretical Cell Yield Achieved from Human- and Computer-directed Subcultures. (A) Human-directed subculture. (B) Computer-directed subculture. Both human- and computer-directed subcultures required 3 serial passages to achieve a theoretical cell yield of 50 million C2C12 cells without exceeding 0.5 confluency.

The overall scheme of the real-time adaptive subculture system is summarized in Figure 1. The automated computer vision system comprised an automated phase-contrast time-lapse microscope that acquired images every 5 min from multiple dishes housed in a heated-stage incubation chamber. These phase-contrast images were subsequently uploaded with Rsync to a server, where computer vision processing was used to identify cells within each image to determine the degree of confluency (Figure 1 and Figure 2). Predictive modeling employing a 2^nd^ order polynomial fit was empirically found to be most suitable for predicting C2C12 growth (Methods S1, Figure S1 and Figure S2) and was used to determine when the level of confluency would reach a user-defined threshold of 0.5 confluency (Figure 1, Figure 3 and Figure 4). 4 hours prior to reaching this threshold, the predictive modeling subroutine alerted the human operator, via text messaging and email to make preparations for cell culture (Figure 1 and Figure 5). In addition, the web-based application facilitated remote monitoring of confluency in individual dishes as well as confluency measurements and predictions via the Internet (Figure 3). When cells reached the threshold for confluency, another email and text message was sent to remind the user (Figure 1 and Figure 5). The cells were then subcultured and the total number of cells was determined (Figure 1 and Figure 6). If the target cell number was achieved, the experiment was terminated (Figure 1). Otherwise, the cells were re-plated at low density for further cell expansion and the process was repeated until the target cell number was reached (Figure 1).

### Evaluation of Confluency

To determine the confluency in a given phase-contrast image (Figure 2Ai), a process known as restoration was applied (Figure 2Aii and 2Aiii) to generate an artifact-free image (Figure 2Aiii). Basic thresholding was subsequently used to obtain a binary image (Figure 2Aiv) consisting of cell (white) and non-cell regions (black), which we have termed as a ‘confluency mask’. Taking into account that several cellular structures such as filopodia and lamellipodia were difficult to distinguish against the image background, this confluency mask was further dilated by a factor of 8 (Figure 2Av). The resultant confluency mask (Figure 2Avi, green) was overlaid on top of the original image and showed good correspondence with regions containing cells. To quantify this correspondence, phase-contrast images at different levels of cell density were manually segmented using a red marker pen to trace the outline of cells (Figure 2Bi). The cell tracings were digitized and filled in to generate confluency masks (Figure 2Bii). The human-generated confluency mask (Figure 2Biii, Red and yellow regions) shows good correspondence with the computer-generated confluency mask (Figure 2Biii, green and yellow regions) with overlapping regions highlighted in yellow (Figure 2Biii, true positive) against the background (Figure 2Biii, black regions, true negative). Regions highlighted only in green and red were considered false positives and false negatives, respectively (Figure 2Biii). Some discrepancies between the human- and computer-generated confluency masks were observed, particularly for large, well-spread out cells (Figure 2Biv). The precision of the computer-generated confluency measurement was determined to be 0.791±0.031 with a recall of 0.559±0.043 (Table 1).

**Table 1:** Confluence Measurements from Computer-Directed Subcultures.

	± SEM
Difference between Time-to-Estimated Threshold for Confluency and Actual Threshold for Confluency^a^ (hours)	4±0
Average Confluency at Time of Notification^a^	0.499±0.003
Precision^b^ (TP/TP+FP)^c^	0.791±0.031
Recall^b^ (TP/TP+FN)^c^	±0.043

a n = 3 independent experiments, ^b^ n = 3 phase-contrast images, ^c^ TP  =  true positive; FP  =  false positive; FN  =  false negative.

### Performance of Real-time Adaptive Subculture System

To determine if the performance of the real-time adaptive subculture system was similar to that of an experienced human operator, a set of human- and computer-directed cell expansions were performed. To facilitate such a comparison, time-lapse images from the human-directed cell expansions were processed similarly as the computer-directed cell expansions *a posteriori* to determine confluency in human-directed cell expansions. Both human- and computer-directed subculture data showed similar performance in estimating and predicting when actual confluency (Figure 4A and 4B, red line) was close to the threshold for confluency (Figure 4, blue line). The predictive modeling performed poorly in the initial 200 frames (Data not shown) but progressively became more accurate as more data points were acquired and achieved a variance of close to zero (Figure 4A and b4B green and red line, Figure 4C and Table 1). This enabled an accurate 4 hour prior notification (4 hours±0, σ^2^ = 0) of when the confluency threshold (0.499±0.003) would be exceeded (Figure 5 and Table 1). Using the criteria that C2C12 cells should not exceed a confluency of 0.5, both human- and computer-directed cell expansions required 3 serial cultures to reach a theoretical yield of 50 million C2C12 cells (Figure 6) with an average cell yield of 22.71±1.97×10^4^ cells/dish and 19.92±1.22×10^4^ cells/dish, respectively (Table 2). No significant difference was observed between the average cell yield obtained from human- and computer-directed cell expansions (p = 0.308).

**Table 2:** Average Cell Yield per 35 mm Petri Dish for Human- and Computer-directed Subcultures.

	Number of Cells (x 10^4^ cells per 35 mm Petri dish) ±SEM	
**Human-directed Subculture**		22.71±1.97
**Computer-directed Subculture**		19.92±1.22

**Figure 7:**
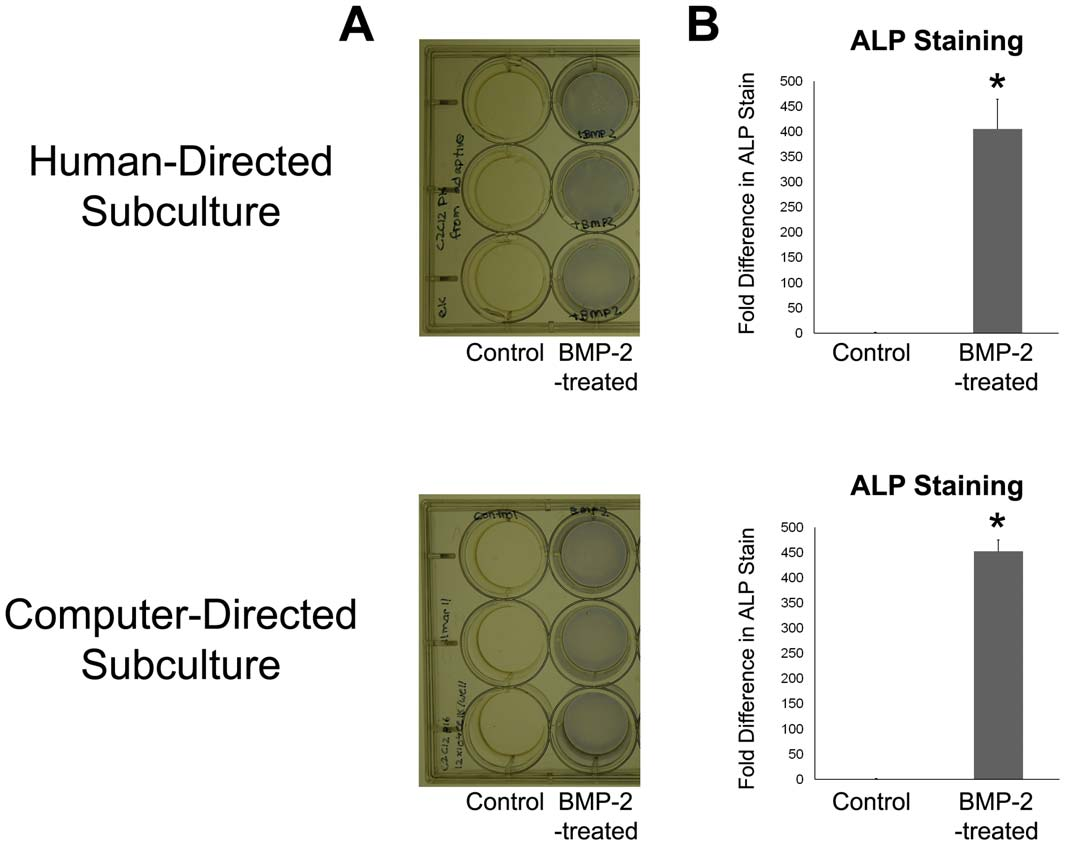
ALP Staining of Human- and Computer-directed Subcultures. (A) ALP-stained plates. BMP-2-treated cells stain positive (blue) for the osteogenic marker, ALP. (B) Quantification of ALP intensity (fold difference compared to control). This shows that cells expanded from both human- and computer-directed subcultures were still responsive to BMP-2-induced ALP expression, indicating that cells were differentiating towards an osteoblast fate.

**Figure 8:**
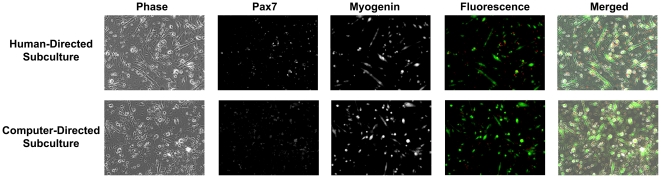
Myogenin and Pax7 Staining of Human- and Computer-directed Subcultures. After 96 h in myogenic conditions, cells that have fused and differentiated towards a myocyte fate (elongated cells containing multiple nuclei) stain positive for the myogenic marker Myogenin (green). Undifferentiated cells stain positive for the stemness marker, Pax7 (red). This shows that cells expanded from both human- and computer-directed subcultures were capable of myocyte differentiation while undifferentiated cells retained their stemness marker.

In addition, human- and computer-expanded C2C12 cells showed increased ALP expression compared to control (p = 0.021 for human-directed cell expansion and p = 0.002 for computer-directed cell expansion) in response to BMP-2 treatment, indicating that BMP-2-treated cells were differentiating towards an osteoblast fate (Figure 7). Furthermore, when grown under low-serum conditions to induce myogenesis, both human- and computer-expanded C2C12 cells stained positive for the myogenic marker, myogenin in multi-nucleated and elongated myotubes (Myogenin, Figure 8) whereas undifferentiated mononuclear cells stained positive for the stem cell marker, Pax7 in the cell nucleus (Figure 8). Together, these results confirmed that both human- and computer-directed expanded cells maintained their stem cell capacity and were capable of undergoing osteogenic and myogenic differentiation under the appropriate conditions (Figure 7 and Figure 8).

## Discussion

Toward the goal of achieving complete automation and consistency for stem cell culture expansions, we have developed an automated computer vision-based system for continuous monitoring and analysis of *in vitro* cell cultures to objectively determine the appropriate time to subculture cells based on confluency measurements (Figure 1). The main impetus for this work is that in the current state-of-art automated cell culture machines are capable of executing the cell passing procedure with high precision and minimal variability [7], however, they lack the ability to make adaptive decisions based on how fast or slow a culture of cells are growing. Furthermore, in manually-operated systems, estimation of confluency by human operators is highly subjective and dependent on the experience of the operator. The confluency measurement algorithm developed here may additionally serve as a useful training aid for new tissue culture users and help to reduce the subjective nature of confluency estimation.

This system employs phase-contrast microscopy as it is a non-destructive imaging modality that is capable of generating high-contrast images of transparent specimens such as cells [26]. Thus, cells can be easily visualized and imaged at high frequency, allowing the user to continuously monitor live cultures without affecting cell viability. Although an image acquisition period of 5 minutes was utilized in these experiments, cells can be imaged at a higher or lower frequency in accordance to experimental requirements, with computer hard disk space and computer algorithm run-time being the only limitations. After image acquisition, the archived data is sent to a server for processing (Figure 2) and 2^nd^ order polynomial curve fitting is used to predict future confluency (Figure 1, Figure 3 and Figure 4). Although the system is designed to be eventually autonomous when combined with a robotic-handling cell system, a human operator can remotely monitor the current and predicted levels of confluency as well as individual fields of view over the Internet (Figure 3). 4 hours prior to reaching a predefined threshold for confluency of 0.5, the human operator is alerted by the computer vision system via email and text messaging (Figure 5) to facilitate preparations such as warming media and various other reagents for cell culture. An additional reminder is sent when the predefined threshold for confluency is reached (Figure 5).

Although a standard computer with a dual core processor and 2 GB Ram is sufficient for implementing the confluency measurement and predictions on a local machine, a server (cloud computing cluster) was utilized so that multiple experiments can be conducted in parallel from different microscope computers or locations. This capability was recently demonstrated for single-cell tracking experiments using image data acquired simultaneously from Tokyo, Japan and Pittsburgh, USA (Intel Developer Forum 2010, California, USA; data not shown). Rsync was chosen for facilitating file transfers because it utilizes a delta-transfer algorithm, which reduces the amount of data sent over the network by sending only the differences between the source files and the existing files in the destination [24]. The algorithms employed in this automated computer vision-based system are efficient and can process one set of images (12 phase-contrast images) prior to acquisition of the next set (within 5 minutes), enabling real-time monitoring and analysis of cell cultures. Although a high image acquisition rate is unnecessary for predicting confluency 4 hours ahead of time (Figure S2), it was used to demonstrate that accumulated photonic energy from a high image rate is non-detrimental to cell growth and that it would be feasible to eventually incorporate it into a real-time cell tracking software to monitor actively migrating and proliferating cells (Intel Developer Forum 2010, California, USA; data not shown), a process requiring high image rates. Furthermore, although less frequent data acquisition is adequate under normally progressing culture conditions (Figure S2), more frequent acquisition times would enable earlier identification of problems in a given culture, which may facilitate earlier interventions to mediate against possible loss of expensive or unique stem cell culture populations.

Confluency, which is the percentage of the surface area in the cell culture vessel covered by cells, is traditionally used by cell biologists as a convenient indirect way to estimate the number of cells in a cell culture vessel. Thus, measuring confluency of an image area occupied by cells is an appropriate measure for estimating cell growth rates. The confluency algorithm utilized here segments cells on the basis of thresholding on the local intensity value of image pixels, a frequently applied methodology used in image segmentation. However, phase-contrast microscopy images have several characteristic halo and shade-off artifacts that arise as a result of technical limitations in the optical assembly of a phase-contrast microscope [26]. To improve segmentation results, a restoration process was applied to remove the halo and shade-off patterns to reconstruct an artifact-free image [25]. The output of the confluency algorithm has good correspondence with manually annotated confluency (Figure 2B) with a precision of 0.791±0.031 and recall of 0.559±0.043 (Table 1). Precision and recall were used to assess the performance of the computer-generated confluency measurements because they are widely employed performance metrics used for pattern recognition algorithms. In general, a precision and recall close to 1.0 indicates good recognition performance. Although some discrepancies between the human- and computer-generated confluency masks were observed, these errors stemmed largely from large, well-spread out cells whose cellular processes were difficult to discern from the image background (Figure 2Biv), resulting in low recall (0.559±0.043) owing to higher false negatives (Figure 2Biv and Table 1). However, the precision (0.791±0.031) of the computer-generated confluency measurements is fairly high and these discrepancies in confluency measurement ultimately did not adversely impact C2C12 cell growth and differentiation (Figure 6, Figure 7 and Figure 8). Given that it is possible to segment cells with ease using computer vision technology, future improvements to the algorithm will incorporate additional measures such as cell density and assessment of cell shape, which may further inform cell culture decisions such as determining the percentage of differentiated cells in culture.

To make confluency predictions, several data fitting models were empirically tested using previously acquired time-lapse phase contrast microscopy images of C2C12 cells and it was determined that that a 2^nd^ order polynomial model produced the best curve fit (Figure S1) and that approximately 200 frames were required before prediction becomes reliable and the variance becomes lower than 0.001 (Figure 4). A 4 hour advance notification was used in conjunction with the prediction model because this was a sufficient time window to allow for planning if personnel were on site, or for a reasonable transport timeframe back to the laboratory, and/or preparation for subculture. Although a 2^nd^ order polynomial was empirically found to be most suitable for predicting C2C12 cell confluency (Figure S1) and adequate for expanding C2C12 cells (Figure 7, Figure 8 and Table 2), this growth model may prove inadequate when culture conditions are altered or when different cell types are used. This is due to the sensitive nature of cells to their environments, which can be severely impacted by variability arising from cell handling, cell passage number, undefined media components such as fetal bovine serum as well as differences in growth and cell spreading rates when considering different cell types. For example, a population of cells that has been recently thawed from liquid nitrogen storage will display a slower rate of growth when compared to the same population after several passages. Mathematical models such as a 2^nd^ order polynomial do not take perturbations in cell culture conditions into account and will exhibit poor performance when used under such scenarios. We have recently developed a data-driven approach [23] to build a confluency prediction model specific for C2C12 cells based on previously acquired data. As long as cell culture conditions are constant, this model is able to predict confluency at least 8 hours in advance with a low error rate [23] and this will be incorporated into subsequent work.

This system was successfully used to direct the expansion of C2C12 cells with the confluency threshold set at 0.5 (Figure 3, Table 1 and Table 2). C2C12 cells are utilized as a paradigm stem cell population because they are a multipotent cell line that has been previously shown to differentiate into cells of the musculoskeletal system [27], [28], [29], [30], [31]. In addition, C2C12 cells are sensitive to the level of confluency and must not be allowed to become confluent, otherwise, cells will spontaneously fuse and deplete the progenitor or stem cell population. Thus, this cell line serves an excellent model for testing our real-time adaptive subculture system. Both human- and computer-directed subculture experiments each required 3 serial passages to theoretically reach a target number of 50 million cells (Figure 6) and showed no significance difference (p = 0.308) in average cell yields per dish (Table 2).

Following cell expansion, C2C12 cells were differentiated towards osteogenic and myogenic fates to confirm that cells retained their capacity to differentiate into multiple cell types such as osteoblasts and myocytes (Figure 7 and Figure 8). In addition, undifferentiated mononuclear cells were shown to retain their stem cell identity as evidenced by positive staining for the stemness marker, Pax7 (Figure 8).

It is interesting to note that although the computer-directed cell expansion had lower variability in terms of cell yield compared to the human-directed cell expansion (Table 1), it is not drastically different. This result may stem from the limitation that only 12 fields of view were utilized in this experiment. Although attempts were made to ensure that the 12 fields of view were representative of culture conditions in each of the 4 dishes, it is possible that sampling a larger number of fields of view may lower the variability observed from cell yield. In addition, the use of a hemocytometer for manual cell counting may have also contributed to an increase in cell yield variability due to sampling error.

Given that stem cell manufacture for different applications, including large-scale production for multiple patient use and small-scale production for autologous use, requires extensive *ex vivo* handling and expansion of cells from various tissue sources [32], a QA/QC system is vital to ensure robust production of cells that are of consistent quality. Although our current system does not incorporate warning alarms to alert users of potential problems associated with cell growth, such problems are easily noticed when overall cell confluency decreases. In such scenarios, individual fields of view can be observed remotely to identify problems (Figure 3). Future work will incorporate additional algorithms that detect and measure cell behaviors such as mitosis [33], apoptosis and cell fusion to provide a more comprehensive view of cell population behavior for maximizing stem cell growth while minimizing stem cell differentiation. Although the use of a microscope stage incubation chamber limited the number of petri dishes that can be observed at a particular given moment, it provided a representative overview of cell growth and confluency to facilitate manual stem cell production with little to no obvious loss in myogenic and ostegenic potential (Figure 7, Figure 8 and Table 2). In addition, our system can be integrated with existing commercially available robotic technology for handling cell culture flasks, allowing for the confluency of every individual flask or dish to be monitored provided that the time required for imaging the desired number of cell culture vessels does not exceed the image acquisition rate. In scenarios where a large numbers of cell culture vessels must be monitored, multiple instruments and computers may be employed in parallel to decrease the time required for image acquisition and image processing.

In summary, we have developed an automated computer vision-based system for adaptive subculture of stem cells based on confluency. Using mouse C2C12 cells as a paradigm stem cell population, this study demonstrated that both human- and computer-directed cell expansions had similar performance in terms of the number of serial passages required to reach a target cell yield. Furthermore, both human- and computer-expanded cell populations were capable of differentiating towards osteoblast and myocyte fates, indicating that stem cell capacity was not lost during cell expansion. This capability offers an approach to reproducibly expand cell populations and may have applications in the manufacture of clinically-relevant cells and/or their cell-derived products. Future work on this system will move towards complete automation of cell culture and QA/QC along with improved algorithm accuracy.

## Supporting Information

Figure S1 Modeling C2C12 cell confluency by five methods utilizing every data point/frame: (1) 1^st^ order polynomial (yellow line), (2) 2^nd^ order polynomial (blue line), (3) 3^rd^ order polynomial (green line), (4) logarithmic function (magenta line), (5) Exponential (red line). The computer-generated confluency measurement (black line) and the confluency threshold (grey line) are indicated. The 2^nd^ order polynomial model fits the observed data (actual computed confluence) with the least root mean square error (RMSE). 1 data point/frame is equivalent to 5 min. The data shown were derived from image sequences of C2C12 cells from 3 independent experiments, each with at least 4 replicates (n = 12).
(TIF)

Figure S2 Comparison of C2C12 cell confluency predictions utilizing every data point/frame (blue line) versus every 6^th^ data point/frame (red cross). The computer-generated confluency measurement (black line) and the confluency threshold (grey line) are indicated. Both 2^nd^ order polynomial models fit the observed data (actual computed confluence) with little-to-no difference in root mean square error (RMSE), indicating that every 6^th^ data point/frame is sufficient to make accurate cell confluency predictions. 1 data point/frame is equivalent to 5 min. The data shown were derived from image sequences of C2C12 cells from 3 independent experiments, each with at least 4 replicates (n = 12).
(TIF)

Methods S1 Calculation of Root Mean Square Error (RMSE) for comparing different confluency prediction models.
(PDF)
