# Supplementary material for: An Engineered Approach to Stem Cell Culture: Automating the Decision Process for Real-Time Adaptive Subculture of Stem Cells
Source: PLoS One. 2011 Nov 16;6(11):e27672. doi: 10.1371/journal.pone.0027672 (PMC3218005; doi:10.1371/journal.pone.0027672)
Supplement: Methods S1 — Calculation of Root Mean Square Error (RMSE) for comparing different confluency prediction models. (PDF) [file pone.0027672.s003.pdf]

An Engineered Approach to Stem Cell Culture: Automating the Decision Process for Real-Time Adaptive Subculture of Stem Cells

Dai Fei Elmer Ker[1,4], Lee E. Weiss[4,5], Silvina N. Junkers[5], Mei Chen[6], Zhaozheng Yin[5], Michael F. Sandbothe[3,5], Seung-il Huh[5], Sungeun Eom[5], Ryoma Bise[7], Elvira Osuna-Highley[4], Takeo Kanade[4], Phil G. Campbell[1,2,4]

[1]Department of Biological Sciences, [2]Department of Biomedical Engineering, [3]Department of Computer Science, [4]Institute for Complex Engineered Systems, [5]Robotics Institute, Carnegie Mellon University, Pittsburgh, PA 15213, [6]Intel Labs, Pittsburgh, PA 15213 [7]Dai Nippon Printing, Tokyo, Japan

Address correspondence to: Phil Campbell, PhD, 1213 Hamburg Hall, 5000 Forbes Ave, Carnegie Mellon University, Pittsburgh, PA 15213. Fax: 412-268-5229 E-mail: pcampbel@cs.cmu.edu

Supplementary Methods

$$\mathrm{RMSE} = \sqrt{\frac{\sum_{i=1}^{N} Error_i^{\,2}}{N}}$$

where

Error = Actual calculated confluency - prediction confluency

N = Number of frames

Methods S1. Calculation of Root Mean Square Error (RMSE) for comparing different confluency prediction models.
